# Can imprinting play a role in the response of *Tetrahymena pyriformis* to toxic substance exposure?

**DOI:** 10.1093/eep/dvw010

**Published:** 2016-07-04

**Authors:** František Ništiar, Oliver Rácz, Marek Brenišin

**Affiliations:** ^1^ Department of Pathological Physiology, Faculty of Medicine, P. J. Šafárik University, Košice, Slovak Republic

**Keywords:** epigenetic imprinting, cadmium, dichlorvos, ochratoxin, Tetrahymena

## Abstract

Among protozoa, *Tetrahymena pyriformis* is the most commonly ciliated model used for laboratory research. All living organisms need to adapt to ever changing adverse conditions in order to survive. This article focuses on the phenomenon that exposure to toxic doses of the toxicants protects against a normally harmful dose of the same stressor. This first encounter by toxicant provokes the phenomenon of epigenetical imprinting, by which the reaction of the cell is quantitatively modified. This modification is transmitted to the progeny generations. The experiments demonstrate the possibility of epigenetic effects at a unicellular level and call attention to the possibility that the character of unicellular organisms has changed through to the present day due to an enormous amount of non-physiological imprinter substances in their environment. The results point to the validity of epigenetic imprinting effects throughout the animal world. Imprinting in *Tetrahymena* was likely the first epigenetic phenomenon which was justified at cellular level. It is very useful for the unicellular organisms, as it helps to avoid dangerous molecules more easily or to find useful ones and by this contributes to the permanence of the population’s life.

## Introduction

*Tetrahymena pyriformis* is a ubiquitous, free-living freshwater ciliate whose physiology and biochemistry has been extensively studied [1]. Its cultivation in the laboratory is very simple and inexpensive. At pH between 6.5 and 7.0 and at temperature 25°C, its reproductive cycle is very short [2, 3]. Under these conditions *Tetrahymena* create up to 8 generations per day, 56 in a week, 240 in a month, and almost 3000 in a year and therefore is an appropriate model for epigenetic studies allowing the simultaneous study of several subjects and their progeny during many generations [4, 5]. In studies of hormone-induced imprinting (term *imprinting* is not exact here, it is used with respect to predecessing works. Probably better equivalent should be methylation-pattern change with followed activation or suppression of genes and *imprinting* is used here in this way) in relation to receptors they were commonly followed up to 1000 generations [6, 7].

In addition to these advantages, members of the genus *Tetrahymena* have other advantages, e.g. their cultures are highly sensitive to growth conditions, various chemical substances in their environment and also to different external physical factors [8, 9].

The sensitivity of *T.pyriformis* to different external factors and their high reproduction rate means that they are a reliable bio-assay model to determine the harmful effects of hazardous chemicals in the environment [10, 11], to monitor industrial and agricultural contaminants, to determine the presence of toxic substances of biological origin in the natural resources, etc. [12].

In addition, *Tetrahymena* as a unicellular animal model has enormous potential of application for a wide range of pharmacological, molecular biological, genetic, immunotoxicological and other experimental studies. They can replace experiments on higher animals for the study of different functions as cell growth arrest, inhibition of respiration and metabolism, synthesis and degradation of specific molecules, etc. [13, 14]. *Tetrahymena* plays an important role in a large number of impressive new molecular genetic technologies and research in the field of functional genomics and provides valuable insights into how these findings are extrapolated to the human genomics [6, 15–17]. *Tetrahymena* was used also in two Nobel-prize winning experimental series; namely in studies of self-splicing character of RNA [18, 19], in discovering the function of telomers and telomerase [20] and contributed to more pioneer studies as (description of lysosome and peroxisome; first isolation of motor protein dynein; (iii) description of somatic genome rearrangement; (iv) introduction of artificial synchronization of cell cycle; (v) discovery of self-splicing RNA; (vi) discovery of the function of histone acetylation, and many other.

In our previous study [21], we proved that *T.pyriformis* W is a suitable model for the study of environmental and industrial pollutants and biological toxins. In this study, we found that cells surviving exposure to LC_50_ of various toxic substances became more resistant to them. The aim of this study was to determine the number of cell generations after exposure to some toxic substances, which are able to keep this “cell memory” of higher resistance.

## Results

In Table 1 we present the results of cytotoxic effects of the tested toxic substances expressed as LC_50/2__ __h_.

**Table 1:** LC_50/2 h_ expressed as % viable *T. pyriformis* W cells

Characteristic	C	OA	DVPP	Cd
Mean	99.84	49.85	49.57	49.29
S.D.	1.17	4.47	3.44	2.67
Maximum	102.00	56.00	54.30	53.30
Upper quartile	100.55	54.48	52.50	51.20
Lower quartile	98.88	44.98	45.98	46.98
Minimum	98.00	44.00	45.00	44.00
Range	4.00	12.00	9.30	8.30
Variation coeff.	0.01	0.09	0.07	0.05
*P* against C		<0.001	<0.001	<0.001

LC_50/2 h_ = 50% lethal concentration after 2 h exposure; C = untreated control; OA = exposed to ochratoxin A (3.2 mg/l); DVPP = exposed to dichlorvos (9.6 mg/l); Cd = exposed to cadmium (24 mg/l); *n* = 10 in all groups. Mean values are inferred from regression lines.

Based on these data we confirmed the values of LC_50/2__ __h_ for the protozoan *T.pyriformis* W for the applied toxic substances found in our previous experiments [21]. The largest variation coefficient was detected for exposure to ochratoxin A.

**Figure 1:**
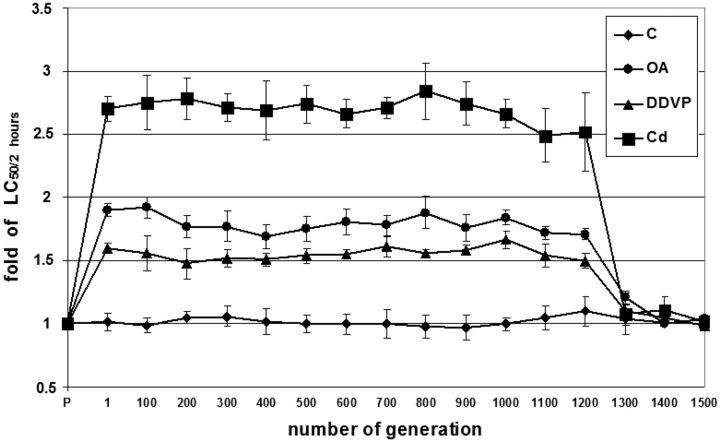
The time course of LC_50/2 h_ after exposure from parental generation to 1500th generation of *T. pyriformis* W. LC_50/2 h_ = 50% lethal concentration after 2 h exposure; C = untreated control; OA = exposed to ochratoxin A; DVPP = exposed to dichlorvos; Cd = exposed to cadmium; *n* = 10 in all groups

After exposure to toxic substances there has been a significant increase in resistance of protozoa against the toxic substances. Values of LC_50/2__ __h_ increased significantly already in the first generation (Table 2 and Fig. 1). LC_50/2__ __h_ after exposure to ochratoxin A in the F1 generation increased 1.9 times, corresponding to a concentration of ochratoxin A 6.08 mg/l, after exposure to dichlorvos increased 1.6 times, which corresponds to a concentration of 15.36 mg/l, and after exposure to cadmium increased 2.7 times, corresponding to a concentration of cadmium 64.8 mg/l.

**Table 2:** Increase in LC_50/2 h_ for further generations of *T. pyriformis* W expressed as multiples of the LC_50/2 h_ used in the first exposure to the same toxic substance as in the parental generation

Characteristic	C	OA	DDVP	Cd
Mean	1.02	1.79	1.56	2.69
S.D.	0.03	0.07	0.05	0.10
Maximum	1.10	1.92	1.67	2.84
Upper quartile	1.05	1.86	1.59	2.75
Lower quartile	1.00	1.74	1.52	2.66
Minimum	0.97	1.69	1.48	2.49
Range	0.13	0.23	0.19	0.35
Variation coeff.	0.03	0.04	0.03	0.04
*P* against C		<0.001	<0.001	<0.001
*P* against OA	<0.001		<0.001	<0.001
*P* against DDVP	<0.001	<0.001		<0.001

LC_50/2 h_ = 50% lethal concentration after 2 h exposure; C = untreated control; OA = exposed to ochratoxin A (3.2 mg/l); DVPP = exposed to dichlorvos (9.6 mg/l); Cd = exposed to cadmium (24 mg/l); *n* = 10 in all groups. Mean values are inferred from regression lines.

As can be seen in Fig. 1, protozoan *T.pyriformis* W were able to resist higher concentrations of these toxic compounds during more than 1200 generations, and then their sensitivity was returned to the initial (original) level. Protozoa tolerated almost 3-fold dose for exposure to cadmium, almost nearly double for exposure to ochratoxin A, and 1.5-fold for exposure to dichlorvos.

## Discussion

In this study, offspring of protozoa surviving exposure to LC_50_ of three different toxic substances increased their resistance to the appropriate toxic substance very quickly and in a rather uniform way. The increased resistance persisted for ∼1200–1300 generations and then suddenly returned to levels found at their first encounter of with the studied toxic substances. This loss can be explained only hypothetically due to possible rearrangement of epigenetic pattern (e.g. methylation) to the starting one, before encounter with toxic substance, as without further exposition, the gained pattern became redundant.

Recognition system of unicellular organisms is comprised by surface receptors recognizing the presence of harmful molecules. Subsequently, these signals through the signal-transduction pathway activate the defense systems, able to bind, neutralize and break down the toxic substance.

Furthermore, tested toxicants are from the point of view according to their structure complexity three different compounds. Cadmium is a basic metal element and also belongs to heavy metals, dichlorvos is a simple organophosphate and ochratoxin A is a common toxic product of food-contaminating mold.

In higher multicellular organisms’ different cells express different sets of receptors. On other hand, in unicellular organisms must be a complete repertoire of receptors expressed on a single cell. Enormous variety of molecules and stimuli can exist in natural aquatic environment of *Tetrahymena* and therefore it should be also a huge number of exprimed receptors on the surface of a single cell. It is not possible to express all of them simultaneously and any time but this paradox could be explained by the fluid mosaic theory of Koch *et al.* [22]. According to this theory, primitive organisms code only receptor subunits and these are continuously incorporated into the cytoplasmic membrane and subsequently recirculated into the cytoplasm. This leads to the fact that the surface of the cytoplasmic membrane structures recognizing foreign molecules is continuously changing. This dynamic system—if the theory is valid, and membrane turnover is sufficiently fast at a high speed and the organism is able to respond to almost unlimited number of molecules present in a changing environment. The fact that the increased resistance in cells surviving after encounter with toxic substance is higher in simple elements and compounds (such as cadmium chloride) and lower for complicated molecules requiring more complex receptor (organophosphate or mycotoxins) supports the validity of Koch’s hypothesis.

The higher resistance was transmitted by protozoa to the following generations is probably the consequence of imprinting, which in the case of hormone induced imprinting has been already described [5, 6]. There are only few data on fixation of imprinting. It is assumed that these are the earliest epigenetic changes, and it has been demonstrated that DNA methylation or rearrangement of histones in nucleosomes significantly affect expression of the genes and that these changes are inherited for following generations [23–26]. The fact that imprinting is an epigenetic process has been reliably demonstrated in the protozoan *Tetrahymena* [27]. Epigenetic modification is very durable and it can explain why is inherited during many generations. In our experiments, we found out that this imprinting persists over 1200 generations similar to hormonal imprinting, which is inherited by more than 1000 generations [27–31]. Imprinting is very beneficial for individual life of *Tetrahymena* offsprings population and helps to protect against dangerous molecules. Interaction of unicellular organism with damaging agents can be remembered, to form a memory of cell which activates defense mechanisms against the appropriate pollutant [17]. Creating of memory mechanisms is also based on epigenetic mechanisms. Though, cellular memory is insufficient itself for learning. Memory and learning are distinct advantages to organisms living in a changing, but recurrent environment [32] and some answers can be expected in these areas from the new results obtained in the study of epigenetics mechanisms. The concept of cellular memory is important in the study of cell biology and differentiation [33, 34]. This can be especially important for microorganisms that live in an environment that neither changes very quickly, nor very slowly.

The results of our experiments confirm that:

- Toxic substances (ochratoxin A > dichlorvos > cadmium) are suitable ligands to develop epigenetic imprinting in *Tetrahymena.*
- Imprinting developed by toxic substances may persist for more than 1200 generations.
- The duration of the changed responsiveness is not affected by the nature of the imprinter toxic substance (possible reason is stated in the first paragraph of the discussion).

These results are preliminary and reliable explanation certainly requires further study.

## Methods

### *Tetrahymena pyriformis* *S* *train and* *C* *ulture* *C* *onditions*

*Tetrahymena pyriformis* strain W were axenically cultured in PPYS medium (pH 6.8–7.0) containing 0.75% (w/v) Proteose peptone (Difco, Detroit, MI, USA), 0.75% (w/v) Yeast extract (Difco) and inorganic salts [35] at a constant temperature of 28°C. Cultures of *T. pyriformis* were maintained by inoculation of 0.5 ml culture every 24 h (about eight generations) into a 100 ml freshly prepared PPYS medium in 500 ml Erlenmeyer bottles.

### *Toxic* *S* *ubstances*

Ochratoxin A (OA), purity of 98% (Sigma-Aldrich, Calbiochem) at a final concentration in the culture medium 3.2 mg/l, which corresponds to a dose of LC_50/2__ __h_. Dichlorvos (DDVP; 2,2-dichlorovinyl dimethyl phosphate, efficiency 98%, Riedel-de Haën), at a final concentration in the culture medium 9.6 mg/l, which corresponds to a dose of about LC_50/2__ __h_. Cadmium chloride (pure, Sigma-Aldrich) at a final concentration of 24 mg Cd/l which corresponds to a dose of LC_50/2__ __h_. Tested compounds were added from stock solutions so that they have the required concentration in test cultures.

### *Exposure to* *T* *oxic* *S* *ubstances*

Parental generation was exposed with a dose LC_50/2__ __h_ of toxic substance in the logarithmic growth phase (10 parallel samples) for 2 h, in the second series of the similarly in 10 subsequent generations (10 parallel samples) for 2 h, respectively.

### *Determination of LC_50/2_*_* *__*h*_ *in* *S* *urviving* *C* *ells*

In subsequent generations after single and repeated exposure, we investigated the dose LC_50/2__ __h_ in the logarithmic phase of growth in the 2nd generation and in approximately every 100th generation to the 1500th generation. Results were evaluated in cultures repeatedly exposed to different concentrations of the toxic substances (0.5, 1, 2 and 4 times the LC_50/2__ __h_) and the control groups (*T. pyriformis* culture with the addition of pure solvent which were used to dilution of tested toxic substances) after the appropriate number of generations of protozoa. Cell cultures were then incubated at 28°C, without shaking in darkness for 2 h. To determine the number of viable protozoa the samples were stained and fixed (by addition of equal volume of 0.4% trypane blue solution and subsequently 5.0% formalin) and calculated microscopically (Leica ICC50 HD) in hemocytometer (Fuchs-Rosenthal chamber). Toxicity of toxic substances was quantified as a percentage of viable cells (%) compared with the number of cells detected in the corresponding untreated control (100% survival), and determining of LC_50/2__ __h_, i.e. the concentration which causes 50% decrease in cell number, which means halved viability under toxic conditions, compared with the untreated control, respectively. For each tested compound and concentration were evaluated 10 parallel samples.

### *Statistical* *A* *nalyses*

Data shown in figure represent mean ± SD. values. The 50% lethal concentration (LC_50/2__ __h_) were calculated by regression analysis with Microsoft Excel 2000 software [36]. A Student’s *t*-test was used for the statistical evaluation of the data. Significance levels were tested at the *P *< 0.05 level.

*Conflict of interest statement*. None declared.
